# Selective Androgen Receptor Modulators Leading to Liver Injury: A Case Report

**DOI:** 10.7759/cureus.67958

**Published:** 2024-08-27

**Authors:** Michael R Demangone, Karam R Abi Karam, Joshua Li

**Affiliations:** 1 Medicine, College of Medicine, Univeristy of Arizona, Phoenix, USA; 2 Medicine, College of Osteopathic Medicine, Kansas City University of Medicine and Biosciences, Kansas City, USA; 3 Family Medicine, Primary Care, Crossover Health, Tempe, USA

**Keywords:** testalone, drug-induced liver injury, liver cirrhosis, sarms, rad-140

## Abstract

Selective androgen receptor modulators (SARMs) have gained popularity for their alleged ability to selectively target androgen receptors, potentially offering muscle-building benefits with fewer side effects than traditional steroids. However, the safety profile of SARMs, including RAD-140, is not fully understood. This case report presents a 29-year-old male who developed liver injury after taking RAD-140. The patient experienced jaundice and elevated liver enzymes after three months of RAD-140 use. A liver ultrasound revealed hepatic steatosis and a hyperechoic lesion. Symptoms resolved after discontinuing RAD-140. Similar cases of liver injury associated with RAD-140 have been reported, highlighting the potential hepatotoxicity of this SARM. Discontinuation of RAD-140 appears to reverse liver injury, but the long-term effects and risks of SARM use remain unclear. This case highlights the need for caution and monitoring when considering SARMs for performance enhancement.

## Introduction

In contrast to traditional steroids, selective androgen receptor modulators (SARMs) work by preferentially targeting androgen receptors in specific tissues, primarily muscle and bone, to increase lean body mass, muscle bulk, and bone density [1-2]. RAD-140's developers aimed to show a potential advantage by highlighting its ability to stimulate muscle growth at lower doses compared to testosterone, which can also increase prostate weight [1]. In one particular study, no dosage of RAD-140 could stimulate the prostate or seminal vesicles to the same extent as testosterone propionate given at a concentration of 1 mg/kg [1]. The primary androgenic activity of endogenous testosterone is increased through its conversion to 5-alpha-dihydrotestosterone (DHT) by the enzyme 5-alpha reductase in tissues such as the scalp and prostate [3]. This conversion amplifies androgenic effects, leading to side effects like hair loss and prostate enlargement. However, SARMs are designed to selectively bind to androgen receptors in specific tissues, such as muscle and bone, without undergoing this conversion to DHT. Therefore, SARMs can exhibit greater selectivity for muscles and bones, promoting anabolic effects like increased lean body mass and muscle growth while minimizing androgenic effects in the prostate and other non-target tissues [3]. With increases in muscle growth and a supposed lack of adverse effects, companies such as Sarmscombo are promoting these drugs, with phrases such as “build muscle with little to no side effects.” [4]. While uncertain, it is estimated that the prevalence of SARM usage in males and females is 6.4% and 1.6%, respectively [5], with the majority of users obtaining SARMs without consulting a physician [6]. The rise in popularity of SARMs motivated the FDA to make a statement in which they issued official warnings and concerns about their usage [7]. As demonstrated in our case study, SARM usage has been associated with drug-induced liver injury (DILI). This report presents a compelling case of liver injury in a male patient, highlighting the potential dangers of the selective androgen receptor modulator RAD-140. We then delve into a comprehensive review of existing literature, synthesizing and comparing similar cases to build a stronger understanding of RAD-140's hepatotoxic effects across diverse clinical presentations.

## Case presentation

On September 5, 2023, a 29-year-old male presented to the clinic due to discoloration in his eyes noticed by his partner a week earlier. Three months ago, the patient had started taking an over-the-counter SARM, specifically Modern Warrior-RAD cycle 10 mg RAD-140 (Testolone), with a total dosage of 20 mg a day. The patient aimed to bulk muscle mass (gain weight) but instead experienced unintentional weight loss. He stopped taking the SARM three weeks before his visit in response to this. He had no known medical or surgical history and did not take any medication. The patient denied alcohol use or illicit drug use but stated he uses chewing tobacco. 

During the physical exam, the patient was in no acute distress and his vital signs were within normal limits. The patient's BMI was 21.6 kg/m^2^. The patient was alert and oriented to person, time, place, and situation. Mild jaundice of the skin and scleral icterus were noted.

The primary care physician (PCP) conducted an extensive workup to determine the cause of the patient's symptoms. Initial diagnostic tests included a comprehensive metabolic panel (CMP), urinalysis (UA), urine culture, fractionated bilirubin, complete blood count with differential (CBC with diff), iron studies, and quantification of immunoglobulins (IgA, IgG, and IgM). Additionally, tests for specific conditions were ordered: copper levels in random urine, liver-kidney microsomal antibody, anti-smooth muscle antibody, ceruloplasmin, alpha-1 antitrypsin, and antinuclear antibody (ANA). 

Additionally, an ultrasound was performed to visualize the liver and trend liver enzyme levels. Differential diagnoses included toxic injury from medication, viral hepatitis, autoimmune hepatitis, and other potential liver pathologies. Given the complexity of the case, the PCP referred the patient to a gastroenterologist for further evaluation and management. 

The patient had two follow-up visits with the same PCP. During each visit, the PCP monitored liver function by checking CMP and fractionated bilirubin levels to track trends.

The following values are from the patient's last visit with the PCP on September 19, 2023. Glucose was 127 mg/dL (normal range: 70-99 mg/dL), total bilirubin was 11.3 mg/dL (normal range: 0.0-1.1 mg/dL), alkaline phosphatase was 192 IU/L (normal range: 44-121 IU/L), aspartate aminotransferase (AST, serum glutamic-oxaloacetic transaminase (SGOT)) was 92 IU/L (normal range: 0-40 IU/L), and alanine transaminase (ALT, serum glutamic pyruvic transaminase (SGPT)) was 144 IU/L (normal range: 0-44 IU/L). According to the UA report, bilirubin was detected in the urine. Analysis of the bilirubin showed a direct bilirubin of 7.34 mg/dL (normal range 0.00-0.40 mg/dL) and an indirect bilirubin of 3.96 mg/dL (normal range: 0.10-0.80 mg/dL). Table 1 compares the patient's laboratory results during the first diagnosis and subsequent values taken throughout treatment. All other values and results obtained were unremarkable or noncontributing to the patient's current presentation.

**Table 1:** The patient's laboratory values significant to liver cirrhosis during first diagnosis and subsequent testing AST: aspartate aminotransferase; SGOT: serum glutamic-oxaloacetic transaminase; ALT: alanine transaminase; SGPT: serum glutamic pyruvic

	Alkaline phosphatase	AST (SGOT)	ALT (SGPT)	Total bilirubin
	(IU/L)	(U/L)	(U/L)	(mg/dL)
Reference values	44-147	14-20	4-36	< 0.3
9/5/2023	152	62	100	8.8
9/12/2023	169	89	139	10.1
9/19/2023	192	92	144	11.3
9/25/2023	169	94	135	12.2

**Figure 1:**
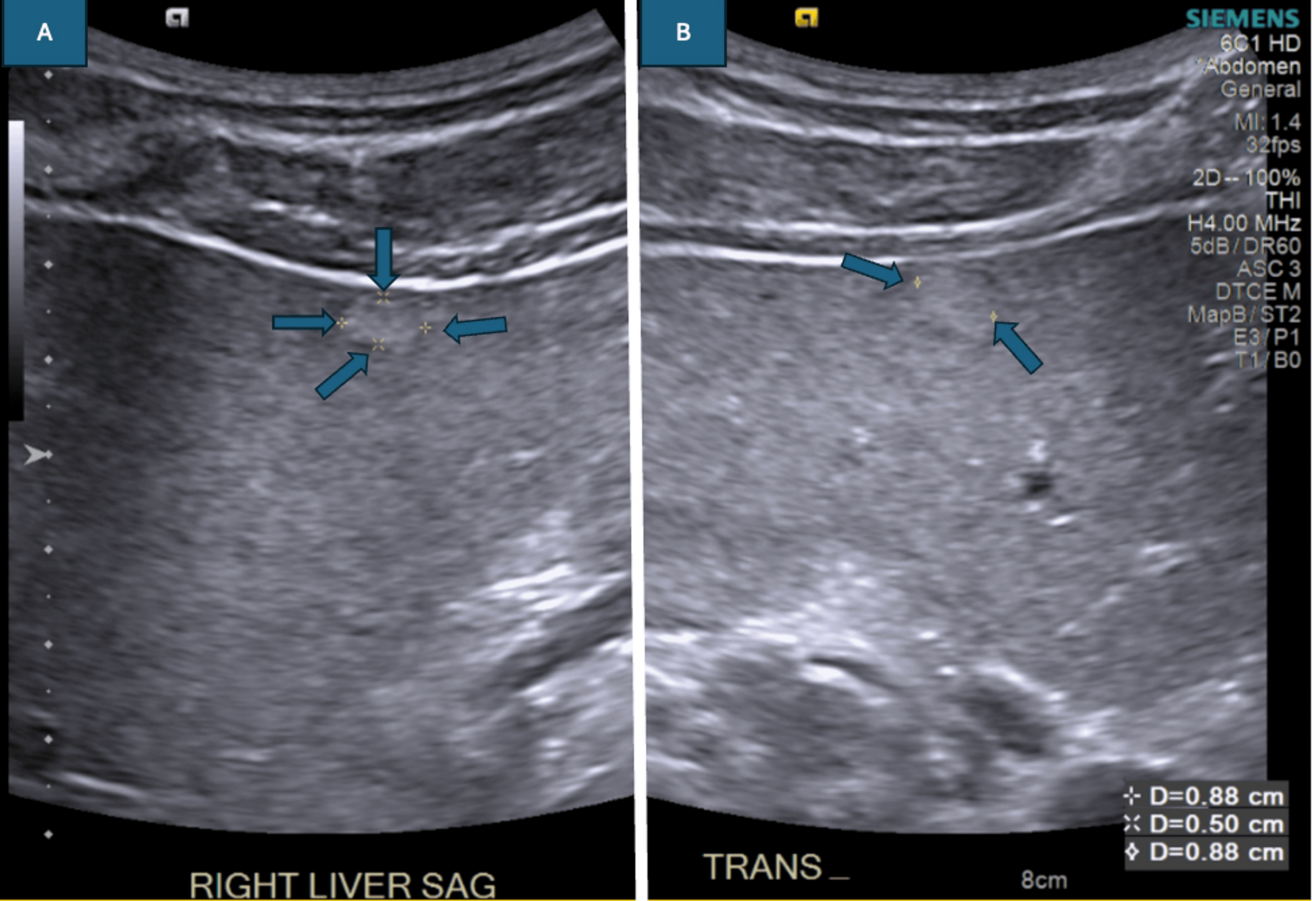
An ultrasound of the abdomen shows a measured lesion A. The hyperechoic right hepatic lesion measures a length of 9 mm and a width of 5 mm; B. The hyperechoic right hepatic lesion measures a height of 9 mm.

An ultrasound (US) of the liver with elastography was ordered. The US of the abdomen (Figure 1) showed increased echogenicity and a hyperechoic right hepatic lesion measured at 9 x 5 x 9 mm. There was no detection of stones or wall thickening in the gallbladder. The US showed hepatic steatosis and F1 fibrosis.

The GI physician evaluated the patient with concerns about elevated liver function tests and suspected DILI secondary to supplement use. The patient underwent a hepatic function panel, with the potential for a liver biopsy if results do not improve. The risks and benefits of a biopsy were discussed, and the patient expressed understanding. The patient’s chronic liver disease workup was largely unremarkable, with imaging showing no biliary obstruction but revealing hepatic steatosis and mild fibrosis (F1). The patient was advised to avoid all supplements, herbs, and teas unless approved by a healthcare provider and to completely avoid alcohol. For pruritus management, cholestyramine was recommended as needed, along with hydroxyzine as prescribed by the PCP. Additionally, oatmeal-based lotions and avoiding hot showers were suggested. A follow-up appointment was scheduled for one month to reassess the patient’s condition and progress.

On October 15, 2023, the patient contacted his PCP’s office to report the resolution of his symptoms. At that time, the patient indicated that he no longer experienced jaundice or pruritus. No additional follow-up appointments were scheduled, and there have been no further reports or updates from the patient since then. 

## Discussion

Selective androgen receptor modulators are increasingly popular among young athletes, typically males, seeking to enhance physical performance or aesthetics while minimizing the risks associated with other forms of enhancement, such as testosterone supplementation [8]. While SARMs are believed to be a safer option for boosting muscle, adverse events have been reported.

As with the patient reported in this case, the use of SARMs has been linked to acute liver injury. While causation cannot be proven between the usage of SARMs and liver injury in our patient, the timing of illness combined with the relative health and young age of our patient leads us to believe that RAD-140 was the culprit. Drug-induced liver injury is typically divided into two categories: intrinsic and idiosyncratic [9]. Intrinsic DILI refers to predictable liver injury caused by drugs, with well-documented dose-dependent effects. Idiosyncratic DILI is more uncertain, and we see higher variability in clinical presentation along with dosing patterns that cause liver injury. 

Due to the rarity and undocumented dose-toxicity relationship of RAD-140-induced liver injury, our case is more likely to fall into the idiosyncratic category of DILI. RAD-140, also known as Testolone, is orally bioavailable and absorbed through the GI tract, where it undergoes first-pass metabolism in the liver. The drug and its metabolites are primarily excreted through feces, with some excretion in urine. The liver's role in metabolizing RAD-140 may lead to the accumulation of toxic metabolites, potentially contributing to hepatotoxicity. These pharmacokinetic properties suggest that liver injury due to RAD-140 could be due to direct hepatotoxic effects or reactive metabolites, highlighting the need for further research to understand the drug’s specific mechanisms and dose-response relationship.

Diagnosis of idiosyncratic DILI primarily relies on clinical expertise. However, the Roussel Uclaf Causality Assessment Method (RUCAM) can be used to supplement the diagnosis and provide a more objective assessment. Due to the patient’s age, clinical presentation, and lack of history for other causes of acute liver injury, our patient received a RUCAM score of six, which gives a probable likelihood that this patient had a case of idiosyncratic DILI. With a high likelihood of RAD-140-induced liver injury in our patient, we decided to document our case alongside other similar reports to bring attention to this possible adverse effect of RAD-140 usage. 

In comparison to previously reported cases, various presentations exist for patients who reported liver injury after taking RAD-140. We’ve documented seven different cases where patients who were taking RAD-140 experienced liver injury. Table 2 summarizes the cases according to age, sex, presentation when first diagnosed, supplement taken, duration of usage, treatment, and time taken for the symptoms to resolve.

**Table 2:** Summary of the related cases y/o: year-old

Author	Age and sex	Presentation	Supplement of interest	Duration of taking the supplement (weeks)	Treatment	Post-treatment symptom resolution (weeks)
This case	29 y/o male	Jaundice and pruritus	20 mg daily of RAD-140	12	Stop supplements and take cholestyramine	6
Barbara et al. [10]	52 y/o male	Right upper quadrant (RUQ) pain, jaundice, pruritus, and diarrhea	7.5 mg of RAD-140 and 5 mg of LGD-4033	7	Stop supplements and abstain from alcohol	35
Leung et al. [11]	24 y/o male	Diffuse abdominal pain, scleral icterus, pruritus, and jaundice	15 mg daily of RAD-140	5	Stop supplements	22
Ladna et al. [12]	26 y/o male	Nausea, vomiting, severe RUQ pain, and jaundice	Not provided	8	Stop supplements	9
Mohamed et al. [13]	22 y/o male	Dark urine, acholic light gray stool, pruritus, and jaundice	Not provided	16	Stop supplements and take cholestyramine	Not provided
Yaramada et al. [14]	24 y/o male	Diffuse abdominal pain, jaundice, and scleral pruritus	Not provided	20	Stop supplements	22
Bhattarai et al. [15]	21 y/o male	Jaundice and pruritus	Not provided	12	Stop supplements	2

It should be noted that our patient did not take acetaminophen, alcohol, or other known substances that are toxic to the liver. In other cases, authors have documented the concomitant use of substances toxic to the liver, such as acetaminophen or alcohol, alongside RAD-140 supplementation [10, 11]. 

While discrepancies in substance use exist, all patients demonstrated liver injury after ingesting RAD-140, suggesting that RAD-140 is a possible common denominator that may be leading to liver injury, regardless of prior substance use. Patient presentations were fairly similar and showcased typical findings of liver injury such as jaundice, diffuse pruritus, nausea, and vomiting. The most common abnormal laboratory values seen were consistent elevations in AST, ALT, and alkaline phosphatase. In general, at least one of these values was abnormal for most patients; however, there does not appear to be any consistent elevation in one lab value over another. 

Based on our compilation of RAD-140 cases, cessation of the drug generally leads to the resolution of liver injury, with no cited cases of irreversible injury. A key distinguishing factor in our case was the abnormal ultrasound, which showed a right lobe lesion, hepatic steatosis, and moderate hyperechogenicity-findings that are atypical compared to previously reported cases where ultrasounds were unremarkable and did not reveal liver lesions, thickening, or biliary dilation.

The absence of a pre-SARM use ultrasound complicates the ability to attribute causation to RAD-140 for the observed liver lesion. Additionally, there is limited information on typical RAD-140 dosages, and the high dose taken by our patient might be associated with the observed liver abnormalities.

Further research is essential to clarify the dose-dependent toxicity and potential liver damage linked to RAD-140 use. The observed recovery timeline of our patient (six weeks) was notably shorter than in other studies, such as Barbara’s [10], which involved concurrent alcohol use, and Yaramada's study [14], where a patient with no known liver toxins had a prolonged recovery period (22 weeks).

To strengthen the evidence, follow-up imaging is necessary to determine whether the liver lesion resolved after RAD-140 cessation. More information on the lesion's characteristics and resolution status post SARM use would provide valuable insights into the causation and prognosis of RAD-140-induced liver injury.

## Conclusions

RAD-140 is an increasingly popular performance-enhancing drug that, while rare, has demonstrated liver toxicity in several cases. The adverse effects are similar to other liver-related injuries; however, there appears to be no long-term implication as symptoms typically regress upon cessation of the drug. Further research is needed to establish standardized protocols for managing RAD-140-induced liver injury and to identify effective treatments and follow-up strategies. Given the potential for liver injury, future follow-up is crucial. Repeat imaging, such as an ultrasound, may be necessary to assess the resolution of liver abnormalities and ensure that no persistent or new lesions have developed. Additionally, monitoring lipid levels and liver function tests during and after the recovery period may help manage and mitigate any ongoing liver-related issues. In terms of recommended treatments, the primary approach involves discontinuation of RAD-140, which often leads to symptom resolution. 

This case report is not the first example of adverse outcomes occurring after SARM usage, and we do not anticipate it to be the last due to the rising popularity of this performance enhancer. A major concern is the potential for SARM usage to induce irreversible liver damage. Although no cases of this have been reported, we advocate for more research in order to better understand the potential harm and long-term effects of SARM usage.
